# Secondary Typhoid

**Published:** 1897-10-23

**Authors:** 


### Secondary Typhoid.

As there seems to be some confusion in the minds of certain people as to the factors at work in the production of great epidemics of typhoid fever, it may be well to point out how distinct are those by which a sudden and widespread outbreak is set up from those which lead to a prevalence of what we may call "secondary typhoid." Quite irrespective of the fact that a polluted well was found, the suddenness of the attack at Maidstone, its wide spread among those supplied with a certain water, its practical limitation at first to the district using this supply, and its distribution among all classes of the community, were data sufficient to stamp it as a "water epidemic." It must be remembered, however, that although typhoid fever is properly enough described as a water-borne disease, in that its infection is usually swallowed along with water or milk, or other forms of food into which it has gained access by way of water, it is only in quite occasional instances that the disease breaks out in widespread epidemics—only, in fact, when the infection has gained access to a widely distributed water or milk supply. The ordinary and normal typhoid, which prevails more or less continuously all over the country, is due to repeated small and purely local outbreaks.

Insanitary conditions exist in many localities, which may go on for years, undetected and unthought of, until the specific infection of the disease is introduced. When this once happens, however, typhoid fever may continue to crop up again and again in the contaminated spot until cleaner methods of collecting drinking water and less filthy methods of disposing of excreta are introduced. There can be no doubt at all that, although typhoid fever is the result of a microbe which grows in man, that same microbe can maintain its vitality, can take root and can grow, in the filth with which man too often surrounds his dwellings. Introduced perchance by some passing tramp, or by the importation of a case of fever from elsewhere, the germ develops and is washed into drinking water, or in the form of dust is scattered over milk or other food, and thus gains access again to man. A vicious circle is thus set up, and although the cases may only recur at such long intervals of time as to be spoken of as sporadic, still, so long as the microbe can maintain its vitality, the disease may remain truly endemic in the place where it has taken root.

The insanitary conditions required to produce this state of affairs are, unfortunately, common enough, and where they exist all that is wanted to light up a local outbreak is the introduction of the infection. Typhoid fever has thus come to be regarded as the touchstone of sanitation. The most careful local sanitation is helpless against the infection of a great water supply under the control of some other authority; but, where the local sanitation is good, so soon as the infected supply is cut off the fever ceases to spread. Given other conditions, however, given a state of insanitation which only requires the sowing of the seed to make the place a hotbed of disease, the natural history of such an epidemic takes quite a different course. The sudden onset characteristic of a water epidemic is the same, but the waning of the outbreak is not the same. All over the place fresh centres of "secondary typhoid" are set up, and long after the first source of the infection has been removed the disease continues. Typhoid, the touchstone, thus sets its mark upon the place. It becomes then a matter of great interest to inquire what is the condition of the drainage of Maidstone, and to what extent are wells still in use as a source of water supply; for, unless the drainage of the town is in a far better condition than the water supply has proved to be, many local foci of possible infection are likely to have been set up, and there still remains a danger of the disease continuing long after the defects in the water supply have been rectified. It is this "secondary typhoid" which is the real test of the sanitary condition of a town.

